# Creencias-prácticas culturales de pacientes con cuidados paliativos domiciliarios, desde la teoría Leininger*

**DOI:** 10.15649/cuidarte.2729

**Published:** 2023-09-07

**Authors:** Leidy Paola Pérez Sandoval, Astrith Liliana Ortiz Mahecha, Nelson Stiven Celis Sarmiento, Nadia Carolina Reina Gamba, Carolina Colmenares, Lina María Vargas-Escobar

**Affiliations:** 1 Universidad El Bosque, Bogotá D.C, Colombia. E-mail: lperezsa@unbosque.edu.co Universidad El Bosque Universidad El Bosque Bogotá D.C Colombia lperezsa@unbosque.edu.co; 2 Universidad El Bosque, Bogotá D.C, Colombia. E-mail: alortiz@unbosque.edu.co Universidad El Bosque Universidad El Bosque Bogotá D.C Colombia alortiz@unbosque.edu.co; 3 Universidad El Bosque, Bogotá D.C, Colombia. E-mail: nceliss@unbosque.edu.co. Universidad El Bosque Universidad El Bosque Bogotá D.C Colombia nceliss@unbosque.edu.co; 4 . Universidad Nacional de Colombia, Bogotá D.C, Colombia. E-mail: ncreinag@unal.edu.co Universidad Nacional de Colombia Universidad Nacional de Colombia Bogotá D.C Colombia ncreinag@unal.edu.co; 5 Cuidarte Tu Salud. Bogotá D.C, Colombia. E-mail: gerenciaoperaciones@cuidartetusalud.com Cuidarte Tu Salud Bogotá D.C Colombia gerenciaoperaciones@cuidartetusalud.com; 6 Universidad El Bosque, Bogotá D.C, Colombia. E-mail: lmvargase@unbosque.edu.co Universidad El Bosque Universidad El Bosque Bogotá D.C Colombia lmvargase@unbosque.edu.co

**Keywords:** Cultura, Cuidados Paliativos, Conocimientos- Actitudes y Práctica en Salud, Cultura, Palliative Care, Health Knowledge -Attitudes Practice, Cultura, Cuidados Paliativos, Conhecimentos Atitudes e Prática em Saúde

## Abstract

**Introducción:**

La cultura del paciente tiene influencia en la selección de sus tratamientos, las expresiones emocionales, la toma de decisiones y en la forma de comunicarse de los pacientes. Los cuidados paliativos permiten el abordaje holístico a personas y familias en sufrimiento por enfermedades potencialmente mortales.

**Objetivo:**

Describir las creencias y las prácticas culturales relacionadas con la salud en pacientes adultos de un programa domiciliario de cuidados paliativos en la ciudad de Bogotá.

**Materiales y Métodos:**

Estudio cualitativo, de tipo etnografía. Se realizaron entrevistas semiestructuradas virtuales a 9 pacientes requirentes de cuidados paliativos, determinada por saturación teórica. Para el análisis de la información, se consideraron las orientaciones de la teoría de los cuidados culturales de Leininger bajo el modelo del sol naciente. Se realizó la codificación y categorización en el software Nvivo v12.

**Resultados:**

Se obtuvieron 3 categorías principales: prácticas culturales, creencias para mantener la poca salud y aliviar síntomas y experiencias en la adaptación a la enfermedad y muerte y se dio lugar a 13 subcategorías

**Discusión:**

Tras describir y analizar los resultados obtenidos, se observó que los participantes poseen conocimientos sobre prácticas-creencias culturales, que han sido transmitidas de generación en generación y juegan un papel importante en el proceso de la enfermedad

**Conclusiones:**

Se evidenció que existen prácticas consideradas protectoras y otras que generan conductas de riesgo, las cuales deberán reestructurarse, negociarse o preservarse, con el objetivo de fortalecer el proceso de cuidado y así mantener un enfoque culturalmente competente.

## Abstract (en)

**Introduction:**

Patient culture influences treatment selection, emotional expressions, decision making, and patients' communication. Palliative care allows a holistic approach to individuals and families suffering from life-threatening illnesses.

**Objective:**

To describe the beliefs and health- related cultural practices of adult patients in a palliative home care program in the city of Bogotá.

**Materials and methods:**

Qualitative ethnographic study. Semi-structured online interviews were conducted with 9 patients requiring palliative care, until theoretical saturation was reached. For data analysis, the guidelines of Leininger's Cultural Care Theory based on the Sunrise Model were considered.

**Results:**

Three main categories were identified: cultural practices, beliefs about maintaining poor health and alleviating symptoms, and experiences adapting to illness and death, resulting in 13 subcategories.

**Discussion:**

After describing and analyzing the results obtained, it was observed that the participants have knowledge of cultural practices-beliefs that have been passed down from generation to generation and play an important role in the disease process.

**Conclusions:**

It became clear that there are practices that are considered protective and others that create risky behaviors that should be restructured, negotiated, or maintained in order to strengthen the care process and thus maintain a culturally competent approach.

## Resumo

**Introdugáo:**

A cultura do paciente influencia na escolha de seus tratamentos, nas expressóes emocionáis, na tomada de decisóes e na forma como os pacientes se comunicam. Os cuidados paliativos permitem uma abordagem holística para individuos e familias que sofrem de doengas que ameagam a vida.

**Objetivo:**

Descrever as crengas e práticas culturais relacionadas a saúde em pacientes adultos de um programa domiciliar de cuidados paliativos na cidade de Bogotá.

**Materiais e Métodos:**

Estudo qualitativo, do tipo etnográfico. Foram realizadas entrevistas semiestruturadas virtuais com 9 pacientes em cuidados paliativos, determinadas pela saturagao teórica. Para a análise das informagóes, foram consideradas as orientagóes da teoria do cuidado cultural de Leininger sob o modelo do sol nascente. A codificagao e categorizagao foram realizadas no software Nvivo v12.

**Resultados:**

Obtiveram-se 3 categorias principais: práticas culturais, crengas para manter a saúde precária e aliviar sintomas e experiencias de adaptagao a doenga e a morte, dando origem a 13 subcategorias.

**Discussao:**

descrevendo e analisando os resultados obtidos, observou-se que os participantes possuem conhecimento sobre as práticas-crengas culturais, que foram transmitidas de geragao em geragao e desempenham um papel importante no processo de adoecimento.

**Condusdes:**

ficou evidenciado que existem práticas consideradas protetoras e outras que geram comportamentos de risco, que devem ser reestruturadas, negociadas ou preservadas, a fim de fortalecer o processo de cuidar e, assim, manter uma abordagem culturalmente competente.

## Introducción

Según la Organización Mundial de la Salud (OMS), los cuidados paliativos se definen como “la prevención y alivio del sufrimiento de los pacientes adultos, pediátricos y sus familias que se enfrentan a los problemas asociados con la enfermedad potencialmente mortal, estos problemas incluyen el sufrimiento físico, psicológico, social y espiritual de los pacientes y sus familias”^1^.

La cultura del paciente es un aspecto importante que debe ser considerado como parte de la atención de los cuidados paliativos. Se reconoce que ésta influye en la forma de comunicación, en la toma de decisiones, en la elección de tratamientos y en la expresión emocional al final de la vida^2^. Es así como conocer las prácticas y creencias culturales de las personas, permitirá a los profesionales guiar las intervenciones, para conservar conductas protectoras e intervenir sobre las conductas de riesgo o con interacción con tratamientos ya establecidos, siendo evaluadas por los equipos de cuidados paliativos.

Cada cultura tiene modelos, valores, modos de vida y símbolos de cuidado, por lo cual la meta de enfermería desde la teoría es brindar cuidados culturalmente competentes, con una atención integral, que contribuya con la calidad de vida^3^. Esta investigación se desarrolló bajo la teoría de enfermería de la universalidad y la diversidad del cuidado cultural y modelo del “Sol Naciente” de Madeleine Leininger, identificando las prácticas culturales y destacando las influencias que ejercen algunos factores como el educativo y económico, las creencias y los estilos de vida, así como factores tecnológicos, sociales y de parentesco, políticos, religiosos y filosóficos sobre la salud. En este sentido, la teoría permite identificar la dimensión cultural y la estructura social de la persona, lo que se convierte en la base para la toma de decisiones y acciones de cuidado.

El objetivo del estudio fue describir las creencias y prácticas culturales relacionadas con la salud de los pacientes adultos de un programa domiciliario de cuidados paliativos en la ciudad de Bogotá.

## Materiales y Métodos

Esta investigación se realizó siguiendo el enfoque cualitativo, etnográfico^4^. Los investigadores abordaron los participantes bajo modalidad virtual. Estos tenían acceso a algún dispositivo electrónico (celular, computador o tablet).

Criterios de inclusión: pacientes con edad igual o mayor de 18 años, pertenecientes al programa domiciliario de cuidados paliativos, con patologías oncológicas y no oncológicas en manejo paliativo, que han expresado su consentimiento bajo la modalidad virtual para participar del estudio. Criterios de exclusión: cuando su condición cognitiva o física no les permitió participar en las entrevistas o si no se contaba con conexión a internet.

La recolección de información se realizó por medio de entrevistas semiestructuradas a nueve pacientes pertenecientes a un programa domiciliario de cuidados paliativos, el tamaño de la muestra se tomó por saturación teórica^4^. La información obtenida se codificó en el software Nvivo v12 y la base de datos fue almacenada en Mendeley Data^5^

El desarrollo de esta investigación se dio durante la emergencia por Covid-19 y se realizó en cuatro etapas secuenciales: 1. Entrevistas semiestructuradas virtual y llamadas telefónicas, 2. Proceso de transcripción línea a línea usando la herramienta Word de Microsoft Office 3. Codificación y categorización: para su realización se combinaron criterios deductivos e inductivos. La fase deductiva, se basó en el marco teórico propuesto por Leininger crearon unas categorías predeterminadas. Por otra parte, el análisis inductivo emergió de la codificación y comparación de códigos y posteriormente se crearon categorías y subcategorías 4. Resultados finales dieron origen a las tres categorías. Cabe mencionar que se consideraron los criterios de rigor científico de credibilidad, auditabilidad, y transferibilidad durante este proceso^6^.

### Aspectos Éticos

El comité de ética de la Facultad de Enfermería de la Universidad El Bosque, aprobó con acta No. 004 2021, No. Código: NUR-2020-143 del 9 de marzo del año 2021, bajo el código de Ética de Enfermería en Colombia^7^ y la resolución 008430 de 1993 del Ministerio de Salud^8^ y se administró el consentimiento informado.

## Resultados

**Tabla 1:** Características Sociodemográficas

Características	n(9)
Género	
Femenino	56,00(5)
Masculino	44,00(4)
Edad	
Entre 20 y 40 años	22,00(2)
Entre 41 y 65 años	44,00 (4)
Mayor de 66 años	33,00(3)
Estado Civil	
Soltero(a)	44,00 (4)
Casado (a)	33,00(3)
Unión Libre	11,00(1)
Viudo(a)	11,00(1)
Nivel de Escolaridad	
Primaria Incompleta	11,00 (1)
Primaria Completa	11,00 (1)
Bachillerato Incompleto	11,00 (1)
Bachillerato Completo	11,00 (1)
Técnico/ Tecnológico	22,00 (2)
Profesional	22,00 (2)
Posgrado	11,00 (1)
Características	n(9)
Régimen de Salud	
Contributivo	89,00(8)
No registra	11,00(1)
Procedencia	
Urbano	89,00(8)
Rural	11,001)
Departamento de Origen	
Santander	56,00(5)
Cundinamarca	22,00(2)
Tolima	11,00(1)
No registra	11,00(1)
Ocupación	
Empleado	11,00(1)
Desempleado	89,00(8)
Ingresos	
Familia	44,00(4)
Arriendo de Inmuebles	22,00(2)
Empresa Propia	22,00(2)
Trabajo	11,00(1)

A continuación, se describen factores de la estructura social descritos por Madeleine Leininger.

### Factores tecnológicos

Los participantes tenían acceso a algún dispositivo electrónico.

### Factores religiosos y filosóficos

El factor religioso tiene gran relevancia entre los participantes, ellos vivencian la espiritualidad desde la religiosidad y filosofía de vida en un ser supremo llamado “Dios” a quien le encomiendan sus afecciones de salud, preocupaciones y anhelos. Algunos participantes según sus creencias esperan alcanzar la trascendencia, cuando hablan de llegar al “cielo”, otros prefieren no pensar en ese final y evitan tratar ese tema. La religión más practicada es la católica, la cual está relacionada con algunos ritos como rezar el rosario, ayunar, orar a ángeles y hacer promesas por sanación.

### Familia y Factores sociales

Se identificaron dos tipos de familias mononucleares y extensas. Los participantes consideraron que el apoyo familiar es vital en el proceso de la enfermedad. Se fortalecieron los lazos afectivos y estos mejoraron la comunicación. La familia reorganiza su estructura y existen cambios de rol; ayuda en el proceso de cuidado de la persona enferma y favorece la satisfacción de necesidades básicas.

### Factores económicos

Se evidencio un cambio en el rol económico y laboral por su situación de salud, algunos participantes empleados, tuvieron que renunciar o sobrellevar las implicaciones que tienen las incapacidades constantes; otros refirieron ser independientes o devengar dinero de actividad propia.

### Factores educativos

El nivel educativo es variable, algunos participantes refirieron primaria incompleta, pregrado y posgrado. Se encuentra la influencia del factor educativo con la práctica de hábitos saludables; también se relaciona con el nivel de conocimiento de la enfermedad y su comprensión. Tener un nivel de educación alto, mostro mayor adherencia a los tratamientos y más interés por buscar alternativas para la “sanación”.

### Valores culturales, creencias y estilos de vida

Los participantes son de distintos lugares de Colombia, principalmente de Cundinamarca, Santander y Tolima, y emigraron a la ciudad de Bogotá en búsqueda de una buena atención. Se observó que muchas de sus costumbres originales se pusieron en práctica con la evolución de su enfermedad como la toma de preparados y uso de plantas.

**Figura 1:**
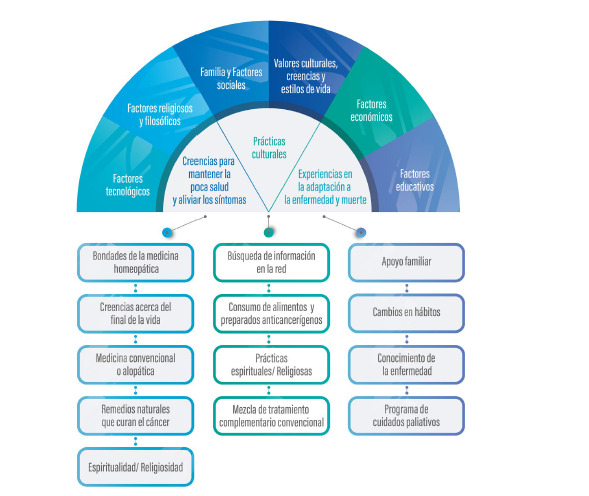
Creencias y prácticas culturales relacionadas con la salud en pacientes adultos en un programa domiciliario de Cuidados Paliativos.

A continuación, se presentan los resultados de las categorías y subcategorías a partir del modelo de Madeleine Leininger. (Figura 1)

### Categoría 1: Creencias para mantener la poca salud y aliviar síntomas

Los participantes manifestaron cómo las creencias pueden favorecer el estado ánimo, creando una actitud positiva en relación con la enfermedad, para mantener la poca salud que tienen y así mismo lograr un mejor control sintomático. Cada participante, se aferra a creencias religiosas, espirituales y culturales, que han sido trasmitidas de generación en generación. Entre ellas, asistir a misa, entregar las preocupaciones y anhelos a ese ser supremo “Dios”, en el que se ha depositado toda la Fé y la esperanza de recibir alivio, misericordia, sanación por medio de plegarias, peticiones u oraciones. Para mantener la salud, se encuentra también el consumo de plantas y frutas con fines terapéuticos.

A continuación, se presentan las 5 subcategorías que surgieron del análisis de la información:

#### 1.1 Bondades de la medicina homeopática

Los participantes buscan ayuda en la homeopatía, dado que en la medicina tradicional no lograron encontrar alivio a sus dolencias y al disconfort generado por los síntomas derivados de las patologías tales como el dolor, espasmos musculares, ansiedad, depresión y estreñimiento:

*“Me recomendaron tomar unas gotas de esencias florales que me ayudan a mantenerme tranquila” (1E).*

#### 1.2 Creencias acerca del final de la vida

Algunos participantes refirieron que es mejor no pensar en el final, sino vivir cada día, además reconocen que el final llegará algún día:

*“No pues que puedo decir...eso ni lo he pensado, solo sé que el día que Dios diga, esto hasta aquí, ese día me voy, pero por ahora estoy contento” (7E).*

*“Yo pienso que a todo el mundo le toca llegar al final de la vida, lo que no sabe uno es cuándo” (3E).*

#### 1.3 Medicina convencional o alopática

Algunos participantes refirieron que el tratamiento de la enfermedad es lo principal, la medicina alopática o convencional, se comporta como el medio para sanación:

*“La única forma de tratar mi cáncer a nivel científico, es por medio de la quimioterapia, además de las terapias de quimioterapia no he hecho más nada” (8E).*

#### 1.4 Remedios naturales que curan el cáncer

Los participantes refieren el consumo de frutas y de plantas medicinales tienen fines terapéuticos, afirman que los conocimientos han sido transmitidos de generación en generación, se observó que el consumo de frutos rojos tales como la mora, agraz y la uva isabelina, se usa para aumentar las “defensas”:

*“La bebida de la vida: es un licuado con moras, remolacha, bueno con muchas cosas de frutos rojos, si incluso le colocan cosas como hígados y mollejas, de pollo también” (1E).*

*Además, algunos participantes refirieron que la enfermedad y algunos tratamientos les generaban síntomas como náuseas, vómito, distensión abdominal; por lo tanto, el consumo de plantas como: tallo de rama de apio, hierbabuena, albahaca y manzanilla les mejoraron estos síntomas:*

*“Manzanilla, albahaca y una rama de apio para cuando uno tiene el estómago inflamado” (9E) “Infusiones de hojas de guanábana, también le hecho una hojita de kalanchoe, un limón congelado rayadito y también sábila” (1E).*

#### 1.5 Espiritualidad/Religiosidad

Los participantes consideraron que la existencia del ser humano está dada por el vínculo existente entre él mismo y “Dios” la importancia del papel de la espiritualidad vivida desde la religiosidad y juega un papel primordial en la propia salud:

*“Pegado al señor y a la santísima virgen María, totalmente, yo soy entregado a él” (4E). Consideraron la oración como opción para mejorar los síntomas generados por la enfermedad.*

*“Toda gira alrededor del señor, de mi esperanza, de mi confianza en él, la misericordia; él me va a sanar y voy a quedar libre del cáncer” (1E).*

### Categoría 2: Prácticas culturales

El cuidado cultural es la relación entre las creencias, experiencias y prácticas de cuidado proporcionadas a pacientes de cuidado paliativo domiciliario con diagnóstico oncológico y no oncológico. A continuación, se presentan las 4 subcategorías que hacen referencia a este apartado.

#### 2.1 Búsqueda de información en la red

En la actualidad cada vez hay más interés por indagar sobre su enfermedad en navegadores y redes sociales, generando una población más informada. Se encontró que los participantes tenían acceso algún dispositivo electrónico y la mayoría de ellos refirieron obtener información mediante el buscador Google:

*“Encontré un chico en internet que decía que para el tumor era bueno tomar agüita de llantén” (2E). “Utilizaba computador cuando me servía, pero ahora para cualquier cosa tengo un buen celular para averiguar todo por este medio.” (1E).*

#### 2.2 Consumo de alimentos y preparados “anticancerígenos”

Los participantes consideraron que el control del cáncer tiene como base realizar cambios de hábitos alimentarios, como la disminución de productos azucarados, grasas, lácteos, café, alcohol y edulcorantes. También se tiene la creencia que el consumo de algunos licuados de frutas con mezclas de hierbas aromáticas favorece el control de la enfermedad.

Las principales estrategias que utilizaron estaban los remedios caseros y cambios en la alimentación. Entre ellos se encontraron: Guanábana, llantén, kalanchoe:

*“Tomar agüita de llantén, infusiones de hojas de guanábana, un licuado de remolacha, manzana, una hojita de Kalanchoe, un limón, también sábila” (1E).*

*“Hojitas guanábana, la tomaba me hacía esa agüita” (1E). “El agua de anamú, la hoja de guanábano, agua de yerbabuena, agua de manzanilla” (2E).*

#### 2.3 Prácticas espirituales/religiosas

Expresaron que en su condición humana la espiritualidad es la manera en que viven el significado y propósito de su vida, su conexión con el momento que están viviendo, con ellos mismos y con los demás, la conexión que tienen con lo sagrado. La mayoría profesa la religión católica y lo importante que es para ellos el apoyo espiritual:

*“Voy a la iglesia y acá en la casa me la paso orando, siempre en la mente y en mi corazón está Dios, por encima de Todo” (2E).*

*“Orar y hablarle, hablarle frente a frente al mismo señor que está en todas partes, osea está en todas partes vivo escuchándolo a uno” (3E).*

#### 2.4 Mezcla de Tratamiento Complementario y convencional

Este tipo de tratamientos no busca reemplazar el uno al otro, por el contrario, se desea potencializar la efectividad de los tratamientos ya establecidos, con productos suplementarios:

*“Mi mamá también está tomando las pastillas de transfer factor de 4life y el riovida, mi médico dice: tómese lo que quiera, igual eso no le va a servir, es la Fé que usted le ponga” (2E).*

Algunos productos de manejo suplementario no tienen suficiente evidencia científica, por ejemplo, existen prácticas como el consumo de productos químicos como el dióxido de cloro, que pudieran ser perjudiciales, pero ante la esperanza de alivio de síntomas y la curación, los participantes asumen los riesgos:

*“Un señor me regaló una botellita de dióxido de cloro y me tomó un centímetro en un vaso de agua, lo puedo tomar dos veces al día o tres veces tiene un saborcito como a límpido o a clorox, espero me ayude” (2E). “Veneno de escorpión azul, es un remedio cubano, los médicos de allí, revisan tu historia y te explican la forma de cómo se prepara, cada cuanto lo tiene que tomar y qué cantidad” (5E).*

### Categoría 3. Experiencias en la adaptación a la enfermedad y muerte

Los participantes refirieron, cómo fue el proceso de adaptación a la enfermedad, ellos encontraron dificultades en el día a día, aunque conocían de la enfermedad por su médico tratante principalmente sobre su diagnóstico. Consideraron que pertenecer al programa de cuidados paliativos ayudó a mejorar su calidad de vida, control de síntomas y atender sus necesidades e inquietudes 24/7. A continuación, se presentan las 4 subcategorías que hacen referencia a este apartado.

#### 3.1 Apoyo Familiar

Para los participantes es de vital importancia contar con apoyo familiar y sentirse acompañados en el proceso de la enfermedad ya que les permitió tener sentimientos de confianza y tranquilidad. La familia se convierte en el proveedor de apoyo emocional y afectivo y realiza la función de cuidador primario.

*“De todos mis hijos tengo apoyo, están muy pendientes de mí” (6E). “Sobre todo mi mamá es mi mano derecha, es quien ha estado pendiente de mí y tengo un hermano, es un apoyo” (8E).*

*“Recibí mucho apoyo y recomendaciones de todo el mundo, porque sale mucha gente a decir cosas sobre la enfermedad” (4E).*

El acompañamiento que reciben por parte de familiares y cuidadores, en el proceso de enfermedad, está centrado en todo tipo de atenciones, entre ellas se encuentra el hecho de preparar y darles “remedios caseros “que están basados en sus experiencias y conocimientos:

*“Mi mamá me dice que el jugo de frutos rojos como el agraz y la mora me sirven para subir mis defensas” (8E).*

#### 3.2 Cambios en los hábitos

Es importante comprender que en el proceso de enfermedad se dieron cambios en la vida cotidiana, los cuales son más notorios en la fase avanzada donde surgen mayores modificaciones en la alimentación y actividades de la vida diaria. En esta etapa la persona realiza cambios de manera voluntaria, entendiendo que es importante mantener su bienestar y considera sacar de su vida hábitos, que bajo su mirada eran poco saludables. Las personas sienten que tienen mayor responsabilidad y compromiso consigo mismas para lograr sobrellevar la enfermedad. Estos cambios se ven determinados por el entorno cultural; por esta razón se puede evidenciar que la alimentación se considera unos de los primeros aspectos que la persona cambia:

*“Ahora soy más consciente de mi alimentación como más pescado, como más carne blanca” “No como carnes rojas, lácteos, dulces” (5E).*

*Por otra parte, la trayectoria de la enfermedad, va dejando cambios en el estado funcional de las personas: “Claro, yo era deportista, salía a correr, brincaba, hacía muchas cosas, salía a jugar fútbol y ahora pues le cambia a uno la vida, ya no soy capaz de nada” (7E).*

*“Ya no puedo hacer tanto, llevo como dos meses sin poder salir a la calle como solía hacer antes” (8E).*

#### 3.3 Conocimiento de la enfermedad

Los participantes refirieron que se les brindó información clara sobre su diagnóstico, evolución y pronóstico, notaban que la enfermedad interfiere en todas las dimensiones de su vida y algunos participantes decidieron confiar en su médico tratante y otros iniciaron la búsqueda de información para aportar en el proceso de cuidado.

Se encontró que el equipo multidisciplinario fue claro con la información y educación que entregó al núcleo familiar, lo que se considera un elemento de gran riqueza en el programa de cuidados paliativos:

*“El cáncer es una enfermedad, células que no salen del cuerpo más que se van multiplicando, el primer médico que descubrió, lo llamó así porque parecía un cangrejo” (1E).*

#### 3.4 Programa de Cuidados paliativos domiciliarios

El pertenecer al programa de cuidados paliativos domiciliarios, les permitió mejorar su calidad de vida, percibieron una atención integral y un acompañamiento activo:

“Los cuidados paliativos son los que se le dan a una persona para mejorar su calidad de vida, un estado en donde se sienta estable, tranquilo, alegre, donde intervienen un psicólogo, ayuda espiritual, los terapistas respiratorios es un grupo, ellos me han dado el apoyo” (7E).

Adicionalmente, los participantes refirieron que al abordaje de cuidados paliativos es integral, se ocupan de la atención al paciente y su entorno, la exploración de los síntomas va más allá del aspecto físico:

*“Ellos están pendientes de tanto física, psicológica y hasta espiritual, de la Fé del paciente y de cómo vive en el entorno familiar, cómo lo cuidan las personas que están con uno, la psicóloga, la trabajadora social, no tenía idea existiera esa forma hasta que me lo formularon y me remitieron de cuidados paliativos” (1E).*

## Discusión

El abordaje de creencias y prácticas culturales relacionadas con la salud, se consideran relevantes para los profesionales de salud, puesto que conocer la perspectiva del paciente, permitirá brindar cuidado culturalmente competente.

Se evidenció que las infusiones de plantas que juegan un papel importante en el proceso de la enfermedad y como parte del tratamiento para el control de ésta o para el control de los síntomas. Lo que muestra que en los participantes existen prácticas culturales principalmente alimenticias, dirigidas al control y tratamiento de la enfermedad. Según lo propuesto por Leininger, los conocimientos que se aprenden y pasan de generación en generación, hacen parte de los factores internos del paciente, en el cual se ven reflejadas las prácticas, creencias y experiencias del conocimiento tradicional, las cuales son importantes para brindar cuidado culturalmente congruente^9^.

Existen otras prácticas culturales que los participantes realizaron, en búsqueda de la “curación” o lograr el control de la enfermedad, en las cuales se pueden asumir algunas conductas de riesgo, como por ejemplo la ingesta de dióxido de cloro. Aunque no existe evidencia científica o autorización para su uso por parte del Instituto Nacional de Vigilancia de Medicamentos y Alimentos (INVIMA), algunos participantes buscan “limpiar” su cuerpo con su consumo. Al contrastar este hallazgo con la literatura, se encontraron estudios que muestran cómo la FDA ha recibido notificaciones de eventos adversos ocasionados por el uso de este producto, causando insuficiencia hepática, diarrea y vómito severo, riesgo de perforación esofágica, falla renal, entre otros, fue considerado una sustancia moderadamente tóxica y peligrosa. Por lo que se concluyó, que no existe evidencia científica que apoye el uso del dióxido de cloro, considerándolo un peligro para la salud^10^^,^^11^. En relación a lo anterior y basado en el modelo de Leininger, se puso en evidencia las herramientas que buscan conservar, negociar o reestructurar aquellos cuidados culturales que generen riesgo para la salud, manteniendo una relación armónica entre la persona y su equipo de salud^12^.

De igual manera se evidenció que la mayoría de los participantes, independiente de la religión que profesan, los participantes encuentran un aliciente cuando ponen en práctica su fe, la oración, el rezar el rosario, hablar con Dios en espacios privados o ir a lugares como la iglesia. Todas estas prácticas buscan que ellos se sientan conectados con la vida y buscar la “Sanación”. En relación con lo anterior, es relevante considerar la atención integral del paciente como parte de los cuidados paliativos, abarcando el área religiosa y espiritual que ayuda no sólo al paciente en el final de vida, sino que también influye en el proceso de toma de decisiones en la salud-enfermedad, para que sea más positivo para el paciente y su familia^13^^,^^14^.En consonancia Leininger desde la dimensión cultural y de la estructura social, se incluye la valoración de factores como la religión (o espiritualidad), parentesco (sociedad), características políticas (ley),entre otros, lo que permite tener una perspectiva más amplia de la persona, entendiendo las particularidades de cada individuo^12^.

Existen otras prácticas encontradas en esta investigación, relacionadas con la búsqueda de información en el proceso de enfermedad este resultado converge con una investigación realizada en España, que demuestra que la búsqueda de información permite mantener un rol más activo en la toma de decisiones sobre la propia enfermedad. También se encontró en la literatura que los pacientes se apoyan en la información de internet, para buscar experiencias de otras personas con diagnósticos similares para comparar, las vivencias, formas de afrontamiento y los tratamientos recibidos^15^, lo que coincide con los resultados de ésta investigación.

De igual forma, los participantes de este estudio, refirieron que no suspendieron el tratamiento convencional, por considerarlo parte importante en el control de su enfermedad, pero incluyeron terapias complementarias. En referencia a los síntomas que traen algunos tratamientos, en un estudio en pacientes con cáncer de cérvix, demostraron que las mujeres recibieron terapias complementarias con la quimioterapia^16^.

Así mismo, los participantes de este estudio hacen cambios basados en el ajuste de la dieta, a través del consumo de suplementos alimentarios, resultado similar a lo encontrado en otro estudio realizado en pacientes mexicanos con cáncer^17^. En relación con lo anterior y según los constructos que propone Magdeleine Leininger, se deben crear las estrategias para orientar el cuidado, basado en prácticas con evidencia científica.

Otro hallazgo relevante, es el pensamiento de los participantes con respecto al final de la vida. Lo que coincide con estudio sobre percepción de los pacientes oncológicos sobre la terminalidad de la vida, trae a colación la importancia de conocer la percepción de los pacientes con cáncer frente al final de la vida; ya que la mayoría de pacientes aun teniendo patologías en fase avanzada, desconocía aspectos como: voluntades anticipadas, qué son los cuidados paliativos y la orden de no reanimar, aspecto que cambiaron después de aclarar estos términos, aceptando de manera más tranquila el hecho de final de vida^18^.

Referente a la categoría de experiencias en la adaptación a la enfermedad y muerte, en este estudio se pudo resaltar la influencia que tiene la familia en el proceso de enfermedad, es de vital importancia para los participantes contar con su apoyo y solidaridad. De igual modo se evidenció que la familia tiene diversidad de formas, comportamientos, acciones y valores. En referencia a lo anterior, durante el proceso de enfermedad los participantes refirieron cambios en hábitos alimentarios, en el rol familiar, social y además pérdidas de su capacidad funcional, lo que afecta tanto a los participantes como a sus familiares, es así como ellos resaltaron la importancia del apoyo familiar; en contraste con otro estudio sobre las relaciones familiares en el contexto de los cuidados paliativos, los resultados son similares a este estudio en cuanto a que se considera que la familia es el soporte para enfrentar los sufrimientos físico, psicosocial y espiritual a los cuales se están expuestos^19^. De igual forma que en el estudio descriptivo observacional sobre Apoyo social a mujeres con cáncer de mama, encontró que la familia es considerada el centro de la sociedad, por lo tanto, es la principal fuente de apoyo en la esfera afectiva y emocional^20^. Por esta razón estos resultados contrastan de manera positiva con los de esta investigación, mostrando que la familia permite disminuir los efectos del proceso de enfermedad.

De igual modo, respecto a la categoría de experiencias, los participantes refirieron que el pertenecer al programa de cuidados paliativos domiciliarios, les brindó acompañamiento, apoyo, sintiendo mayor la seguridad y armonía; puesto que ellos afirman que cuentan con un equipo interdisciplinario, lo que contrasta con un estudio realizado sobre la percepción de las enfermeras de cuidados paliativos, muestra de manera similar que estos cuidados, deben tener un equipo multidisciplinar que aborde las necesidades del paciente y su familia; así mismo resaltan la importancia de prevenir y aliviar el sufrimiento^21^.Por las consideraciones anteriores y basadas en los constructos que propone Magdeleine Leininger, el equipo de profesionales que realiza el proceso de cuidado, deberán conocer al sujeto, sus valores, creencias y prácticas, lo que contribuirá ofrecer cuidado cultural competente^3^.

Para finalizar es relevante mostrar la importancia de la aplicabilidad del modelo teórico de Leininger, que permitió identificar la estructura social y cultural de los pacientes del programa domiciliario de cuidados paliativos.

Así mismo permitió orientar en el análisis de los factores de la diversidad y de la universalidad de los cuidados culturales mostrando que el enfoque cultural es fundamental en las expresiones de cuidado.

Los profesionales de enfermería tienen el reto de conocer el contexto cultural de cada uno de los pacientes, para brindar una atención individualizada y a su vez dar una orientación precisa que logre integrar el conocimiento desde la academia, en donde se muestra la importancia de conocer todos los aspectos sociodemográficos y los factores de Leininger y aquel que es adquirido de manera tradicional^22^.

## Conclusiones

En esta investigación se encontró que la mayor parte de conocimientos, se han transmitido de generación en generación a través de familiares, amigos y conocidos que les habían proporcionado la información, lo que mantiene un legado cultural; en cuanto a las categorías de creencias relacionadas con la salud y el alivio de los síntomas, influyeron en la mejoría del estado de ánimo, creando actitudes positivas durante el proceso de enfermedad.

En la categoría de prácticas culturales, existen unas consideradas protectoras y otras que generan conductas de riesgo, las cuales se pueden llegar a conservar y/o reestructurar en el proceso de cuidado según sea el caso.

En la categoría experiencias en la adaptación a la enfermedad y muerte, evidenció que existen estrategias como: el apoyo familiar, los cambios en los hábitos y pertenecer al programa de cuidados paliativos domiciliarios, que mejoran el afrontamiento a la enfermedad.

Para finalizar el modelo de Leininger permite resaltar el papel del profesional de enfermería el proceso del cuidado, basado en el área socio cultural, lo cual motiva a la búsqueda de actualización y profundización del saber ser, a través de investigaciones sobre las creencias, prácticas y experiencias relacionadas por los pacientes en búsqueda de la sanación o recuperación de la poca salud. Se recomienda en futuras investigaciones profundizar en el contexto cultural, explorando el entorno, canales de comunicación, la afectación biopsicosocial que se da en el choque cultural en relación a los cuidados paliativos.

Como principal limitación fue la obtención de la información durante la pandemia que no permitió incluir la observación participante, que es una herramienta importante en Etnografía.

## References

[1] Organización Mundial de la Salud (2020). Cuidados paliativos.

[2] Luxardo N, Vindrola C (2016). En clave intercultural: Intervenciones en el final de la vida con fundamento en las "diferencias culturales". Revista M..

[3] Briñez K, Muñoz L (2018). Experiencias de cuidado cultural en personas con diabetes y el contexto familiar, con enfoque Leininger. Cultura de los cuidados.

[4] Hernández RS, Fernández C, Baptista P (2014). Metodología de la Investigación Hernández Sampieri.

[5] Leidy Paola Perez Sandoval, Lina María Vargas Escobar, Astrith Liliana Ortiz, Nadia Carolina Reina Gamba, Celis Nelson, Colmenares Carolina (2023). Creencias-prácticas culturales de pacientes con cuidados paliativos domiciliarios, desde la teoría Leininger. Mendeley Data.

[6] Viorato S, Reyes V (2019). La ética en la investigación cualitativa. Revista CuidArte.

[7] Ministerio de Educación Nacional (2004). Ley 911 de 2004. Responsabilidad deontológica en enfermería. Colombia. Congreso de Colombia.

[8] Ministerio de Salud (1993). Resolución número 8430 de 1993.Normas científicas, técnicas y administrativas para la investigación en salud. Colombia.

[9] Bautista J, Piscoya L (2019). Repositorio Institucional UNPRG.

[10] Burela A, Hernández A, Comandé D, Peralta V, Fiestas F (2020). Dióxido de cloro y derivados del cloro para prevenir o tratar la COVID-19: revisión sistemática. Rev Peru MedExp Salud Pública.

[11] Giachetto G, Pardo L, Rodríguez A, Speranza N, Zunino C, Notejane M (2021). Dióxido de cloro y derivados en la prevención y tratamiento de la COVID-19. Arch. Pediatra. Urug..

[12] Alligood M, Tomey A (2018). Modelos y teorías en enfermería. Elsevier Health Sciences.

[13] Tomaszewski A, Oliveira S, Arrieira I, Cardoso D, Sartor S (2017). Manifestares e necessidades referentes aoprocesso de morte e morrer: perspectiva da pessoa com cancer Demonstrations and necessitiesonthedeath and dyingprocess: perspective of thepersonwithcancer. R. pesq. cuid. fundam..

[14] Rudilla D, Soto A, Pérez M, Galiana L, Fombuena M, Oliver A (2018). Intervenciones psicológicas en espiritualidad en cuidados paliativos: una revisión sistemática. MedPaliat..

[15] AbtSacks A, Pablo S, Serrano P, Fernández E, Martin R (2013). Necesidades de información y uso de Internet en pacientes con cáncer de mama en España. GacSanit..

[16] Guzmán J, Alvira D (2021). Efectos secundarios de las terapias oncológicas en pacientes con cáncer de cérvix. Rev cienc..

[17] Navarro M, Reynoso N, De La Piedra A (2018). Surveyonthe use of alternative and complementary medicine in mexican patients with cancer in anoncologyreference center. GacMexOncol..

[18] Comin L, Panka M, Beltrame V, Steffani J, Bonamigo E (2017). Percepción de los pacientes oncológicos sobre la terminalidad de la vida. Rev. Bioét..

[19] Espíndola A, Quintana A, Farias C, München M (2018). Relaciones familiares en el contexto de los cuidados. Rev. Bioét..

[20] Azcárate E, Valle U, Villaseñor R, Gómez A (2017). Apoyo social a mujeres con cáncer de mama en una unidad de medicina familiar de la Ciudad de México. Aten Fam..

[21] Lozano B, Huertas M (2016). Cuidados paliativos, cuidados compartidos. Cultura de los Cuidados.

[22] Hernández M, Alonso M, Suarez D (2020). Prácticas de cuidado cultural para manejo del dolor en el hogar en personas con diagnóstico oncológico, HUSI. Repositorio Javeriana.

